# Asymmetrical positioning of cell organelles reflects the cell chirality of mouse myoblast cells

**DOI:** 10.1063/5.0189401

**Published:** 2024-03-14

**Authors:** Zeina Hachem, Courtney Hadrian, Lina Aldbaisi, Muslim Alkaabi, Leo Q. Wan, Jie Fan

**Affiliations:** 1 Department of Natural Sciences, CASL, University of Michigan-Dearborn, Dearborn, Michigan 48128, USA; 2 Department of Biomedical Engineering, Rensselaer Polytechnic Institute, Troy, New York 12180, USA; 3 Center for Biotechnology and Interdisciplinary Studies, Rensselaer Polytechnic Institute, Troy, New York 12180, USA; 4 Department of Biological Sciences, Rensselaer Polytechnic Institute, Troy, New York 12180, USA

## Abstract

Cell chirality is crucial for the chiral morphogenesis of biological tissues, yet its underlying mechanism remains unclear. Cell organelle polarization along multiple axes in a cell body, namely, apical–basal, front–rear, and left–right, is known to direct cell behavior such as orientation, rotation, and migration. Among these axes, the left–right bias holds significant sway in determining the chiral directionality of these behaviors. Normally, mouse myoblast (C2C12) cells exhibit a strong counterclockwise chirality on a ring-shaped micropattern, whereas they display a clockwise dominant chirality under Latrunculin A treatment. To investigate the relationship between multicellular chirality and organelle positioning in single cells, we studied the left–right positioning of cell organelles under distinct cell chirality in single cells via micropatterning technique, fluorescent microscopy, and imaging analysis. We found that on a “T”-shaped micropattern, a C2C12 cell adopts a triangular shape, with its nucleus–centrosome axis pointing toward the top-right direction of the “T.” Several other organelles, including the Golgi apparatus, lysosomes, actin filaments, and microtubules, showed a preference to polarize on one side of the axis, indicating the universality of the left–right asymmetrical organelle positioning. Interestingly, upon reversing cell chirality with Latrunculin A, the organelles correspondingly reversed their left–right positioning bias, as suggested by the consistently biased metabolism and contractile properties at the leading edge. This left–right asymmetry in organelle positioning may help predict cell migration direction and serve as a potential marker for identifying cell chirality in biological models.

## INTRODUCTION

The cell, as the smallest functional unit of life, exhibits polarity along three axes: apical–basal, front–rear, and left–right. When adhering to a substrate, apical–basal polarity guides the cell to anchor itself to the surface and secrete molecules toward the appropriate side.^1^ In most slow-moving cells, lamellipodia or filopodia form the leading edge, pointing to the anterior of cell migration with the microtubule organizing center (MTOC) polarized at the front side of the cell nucleus.^2^ Since the centrosome is the main MTOC, the front–rear axis of a cell has been widely determined by the displacement of the centrosome in the front of the nucleus, pointing the cell migration direction.^3–5^ Similarly, the polymerization of actin filaments, specific structural proteins, or cellular kinase activities exhibits front–rear polarity inside the cell body.^3^

The left–right polarity of cells had been underexplored until recently, when it was discovered that different types of cells possess varying degrees of chirality.^6^ In addition, it has been observed as left–right asymmetry in various cellular processes such as alignment on micropatterns,^6,7^ migration,^8,9^ tissue morphogenesis,^10,11^ and rotational movements of the cytoplasm,^12^ nucleus,^13^ cytoskeleton,^14,15^ and the cell itself.^16–18^ It is considered an intrinsic property of the cell but can be changed by certain cytoskeletal inhibitors and cellular kinase activators.^6,14,15,19^ Cell chirality can induce the left–right biased cell shape. For instance, it has been found that endothelial cell predominantly polarizes the body centroid toward the right side of its nucleus–centrosome axis, and this cell shape is associated with their strong clockwise chirality.^19–22^ This clockwise chirality of endothelial cells plays an important role in maintaining blood vessel integrity,^19^ while altering it by external factors could lead to severe pathological conditions.^21,22^

Investigating the intricacies of chirality-related phenomena in exploring physiological questions is a challenging endeavor. The primary difficulty arises from the limitations of the specialized bioengineering tools required, and the transient nature of chiral events as physiology progresses.^17,18^ This circumstance underscores the necessity for an internal cellular structure that could reflect the left–right bias of a cell. Thus, it leads back to whether the placement of cell organelles may not only signify the front–rear polarity of cell migration but could also indicate the left–right polarity of a cell. Drawing a parallel to how our bodies house left–right biasedly positioned internal organs within our torso, it can be posited that, albeit on a much smaller scale, the cell could potentially arrange different organelles in a way that exhibits similar left–right biases.

Mouse myoblast cells, known as C2C12 cells, are recognized as a left-biased cell phenotype marked by a distinct counterclockwise cell chirality.^6^ Notably, this inherent chirality can be reversed to clockwise with the treatment of Latrunculin A,^6^ which binds to G-actin in a 1:1 ratio, inhibits actin polymerization and accelerates actin depolymerization.^23,24^ In this study, we delved into the positioning of multiple organelles within a single C2C12 cell and its correlation to cell chirality. The findings illustrate the potential for using organelle positioning as a marker to evaluate the left–right bias of a cell under *in vivo* or *in situ* conditions, where traditional bioengineering tools for studying cell chirality may not be accessible. This provides a look into the nuanced subcellular structure of cells with a predilection for self-organization among their organelles.

## RESULTS

### Latrunculin A induces chirality reversal in C2C12 cells

**FIG. 1.**
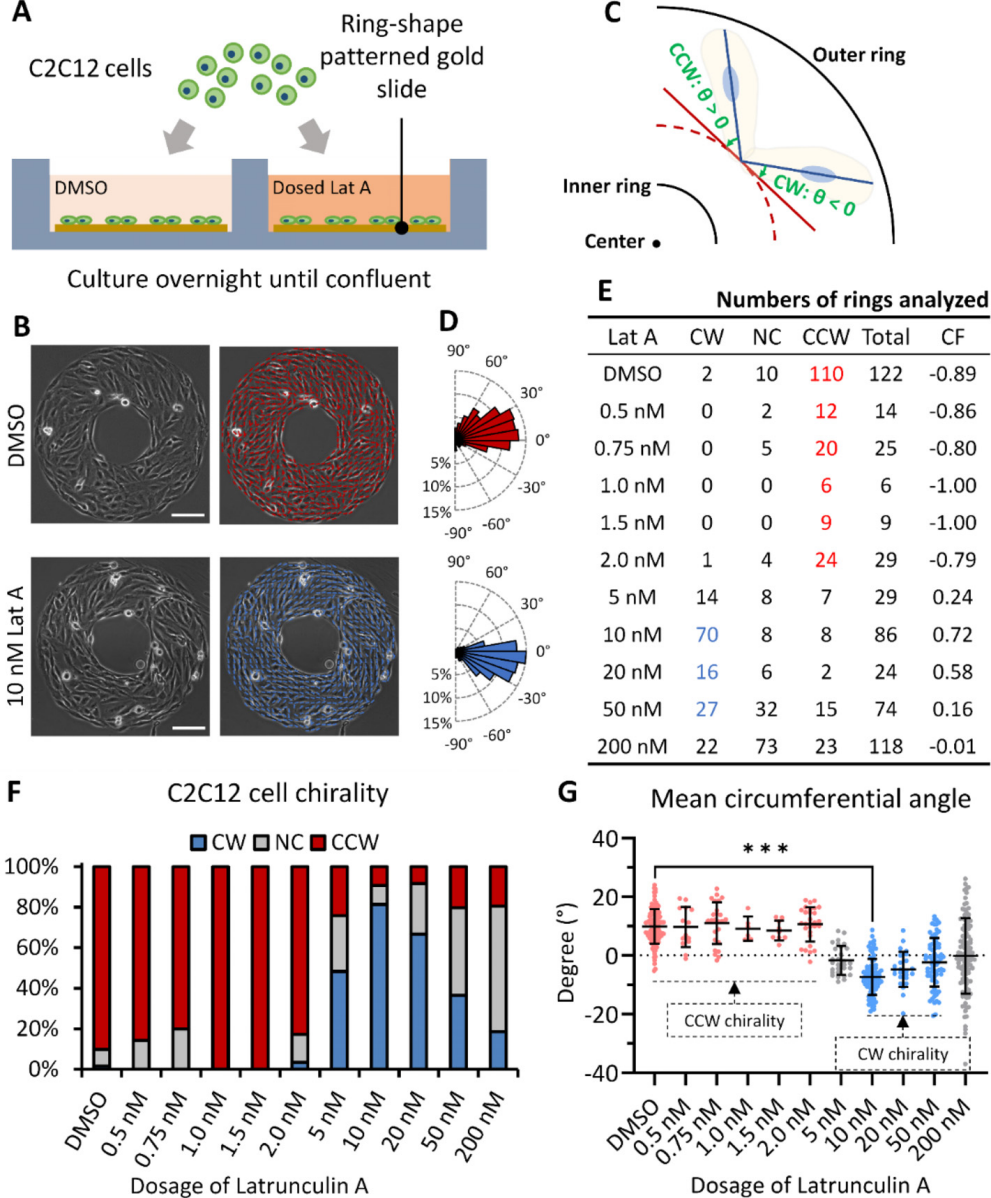
The reversal of myoblast cell chirality under the Latrunculin A treatment. (a) Depiction of the method for culturing C2C12 cells on gold-coated, ring-shape micropatterned glass slides. (b) Representative phase contrast images displaying the formation of counterclockwise micropatterns by C2C12 cells under the control condition, contrasted with the formation of clockwise micropatterns following the treatment of 10 nM Latrunculin A. The red or blue bars, produced by a pre-existing MATLAB program, indicate the alignment direction of each cell on the micropattern. Scale bar indicates 100 *μ*m. (c) Explanation of the circumferential angle of each cell alignment in (b), illustrating its deviation from the circumferential direction (highlighted red) and showcasing its relationship to chirality. (d) Histogram plot of the circumferential angles of cell alignment as shown in (b). The top histogram depicts the control (DMSO) group, and the bottom illustrates the 10 nM Lat A group. (e) Summary of the numbers of clockwise (CW), counterclockwise (CCW), and non-chiral (NC) rings analyzed, including the corresponding chiral factors of C2C12 cells at varying dosages of Latrunculin A. The chiral factor (CF) is calculated as (number of CW rings − number of CCW rings)/number of total rings, with CF = +1 signifying complete CW and CF = −1 indicating complete CCW. The red or blue font indicates dominant bias and significant difference between CCW and CW at P < 0.05 by a rank test. (f) Proportions of CW, NC, and CCW rings of C2C12 cells derived from data in (e). (g) Mean circumferential angle of C2C12 cells across all the ring-shaped micropatterns per group. Data are shown as mean ± SD. The asterisks stand for significant differences between the experimental group and the control: “^*^” indicates P < 0.05, “^**^” indicates P < 0.01, and “^***^” indicates P < 0.001, as determined by one-way analyses of variance (ANOVAs) with the Tukey method between groups.

The chirality of C2C12 cells was assessed by seeding these cells on ring-shaped micropatterns and treating them with varying dosages of Latrunculin A [Fig. 1(a)]. Under the control condition, C2C12 cells demonstrated a strong counterclockwise alignment on the micropattern [Figs. 1(b)–1(d)]. Upon treatment with increasing concentrations of Latrunculin A to 10 nM, the chirality of C2C12 cells is reversed, manifesting a dominant clockwise chirality [Figs. 1(e) and 1(f)]. This switch is quantified by a chiral factor shift from −0.89 under the control condition to +0.72 at 10 nM Latrunculin A treatment [Fig. 1(e)]. Initially, C2C12 cells presented a positive circumferential angle in the control condition, but the angle of cell alignment significantly shifted to negative under 10 nM Latrunculin A treatment [Fig. 1(g)]. These findings corroborate previous reports that a low dosage of Latrunculin A treatment reverses the chirality of C2C12 cells from counterclockwise to clockwise.^6^ In addition, we tested other compounds that interfere with actin filaments or microtubules, and among these, Latrunculin A induced the most significant reversal of C2C12 cell chirality [Figs. S1(a)–S1(c)]. However, elevated concentrations of Latrunculin A above 5 nM significantly increased cytotoxicity. Particularly, at 200 nM, this compound not only caused substantial cell death (Fig. S2) but also disrupted cellular chiral alignment on the micropattern, which significantly affected the chirality results [Fig. 1(g)]. Therefore, to ensure that cells are achievable clockwise chirality with minimal cytotoxic effects, 10 nM Latrunculin A was selected for subsequent studies on the switching of cell organelle positioning.

### The positioning of cell organelles exhibits left–right biases on a T-shape micropattern

**FIG. 2.**
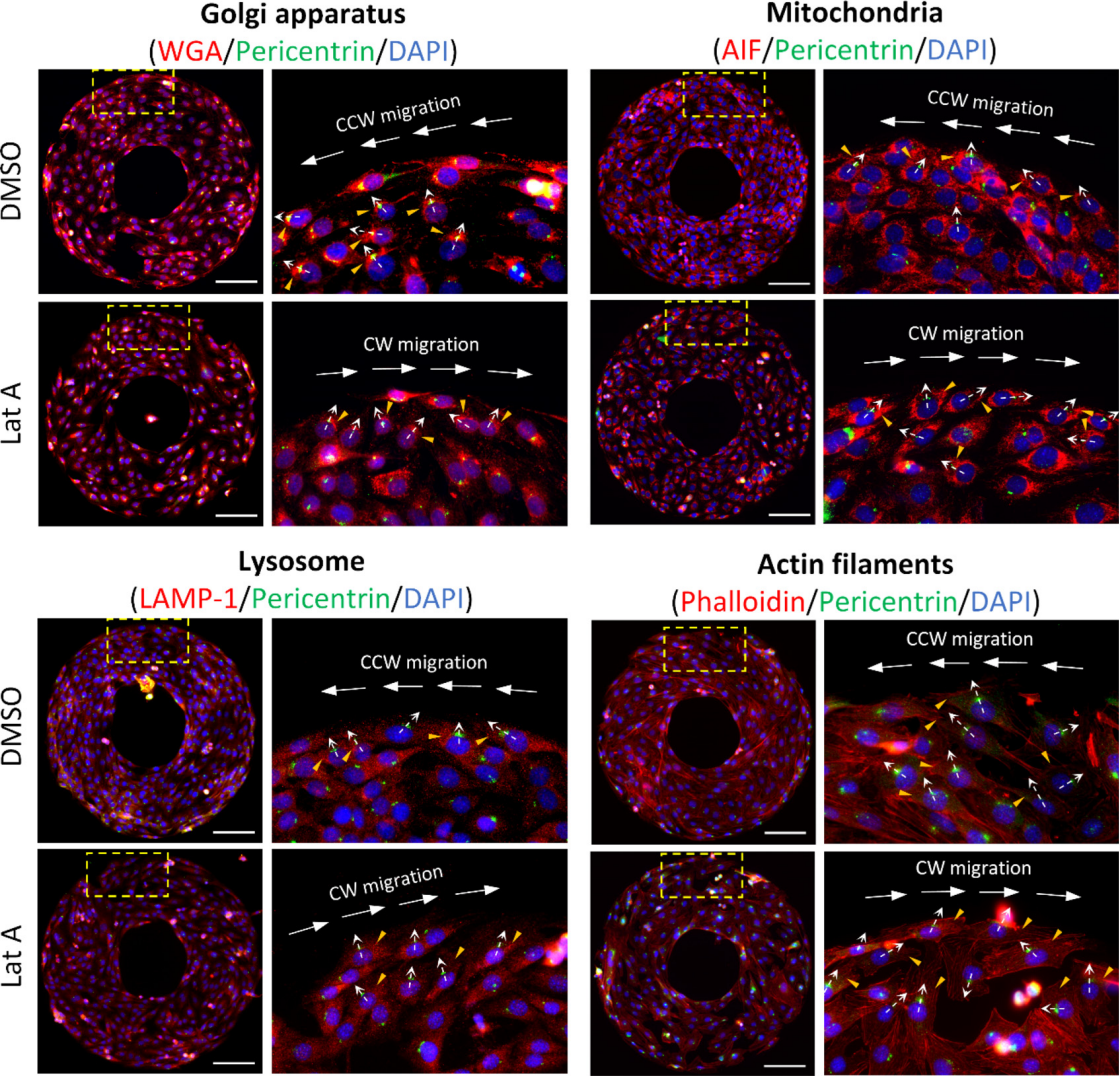
Fluorescent images showing the asymmetric positioning of cell organelles near the border of the ring-shaped micropattern under control and treatment with 10 nM Latrunculin (a). In C2C12 cells, centrosomes and nuclei were labeled with AF488-conjugated pericentrin (green) and DAPI (blue), respectively. The Golgi apparatus was labeled with AF594-conjugated WGA (red), mitochondria with AF594-conjugated AIF (red), lysosomes with AF594-conjugated LAMP-1 (red), and actin filaments with AF594-conjugated phalloidin (red). Higher magnification (40×, right) images of the areas highlighted in yellow dashed boxes (left) were captured to show detail. The dashed white arrows in the zoomed images indicate the polarized nucleus-centrosome axis toward the border of micropattern. Small yellow arrows point to organelles that are significantly positioned asymmetrically along the nucleus–centrosome axis. Scale bar indicates 100 *μ*m.

The nucleus–centrosome axis is widely regarded as the front–rear polarity axis of a cell.^3–5^ Close to the border region of the ring-shaped micropattern, cells are found to be significantly polarized, with their nucleus–centrosome axis oriented toward the outer border.^6^ Additionally, cell chirality has been found associated with the left–right asymmetric positioning of cell organelles relative to this axis in individual cells.^10,18–20^ At the border region of the ring-shaped micropattern, we observed a trend in C2C12 cells under the control condition with counterclockwise chirality; the Golgi apparatus, mitochondria, lysosomes, and actin filaments were preferentially positioned on the left side of the nucleus-centrosome axis (Fig. 2), while the microtubule concentration spots showed no preference (Fig. S3). Conversely, when cells displayed clockwise chirality under Latrunculin A treatment, these organelles were preferentially positioned on the right side of the nucleus–centrosome axis (Fig. 2). This positioning of organelles also appears to correlate with the direction of cell migration at the border of the micropattern.

**FIG. 3.**
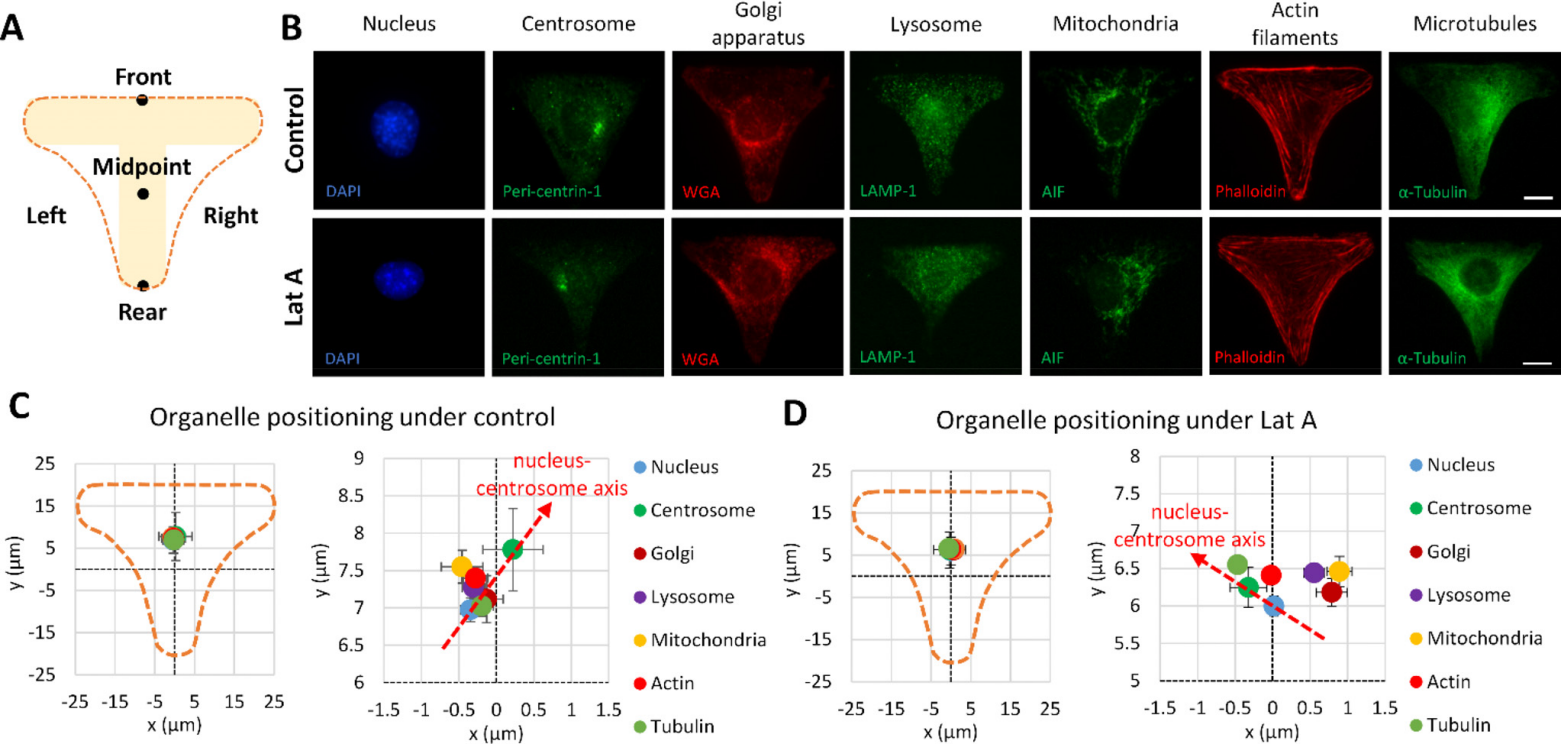
Examination of organelle positioning in a single myoblast cell on a T-shaped micropattern. (a) Schematic representation of the T-shaped micropattern deployed to drive the polarization of a single cell into a triangular cell shape (depicted by a dashed border), illustrating front–rear and left–right orientations following cell expansion. (b) Representative fluorescence images of various organelles labeled with antibodies targeting organelle-specific proteins or with fluorescence-conjugated compounds or labeling dyes in a single cell following a T-shape pattern under the control condition or 10 nM Latrunculin A treatment. The nucleus is marked by DAPI (blue), the centrosome by an antibody against pericentrin-1 (green), the Golgi apparatus by AlexaFluor 594 wheat germ agglutinin (WGA, red), lysosomal vesicles by an antibody against LAMP-1 (green), mitochondria by an antibody against apoptosis-inducing factor (AIF, green), actin filaments by AlexaFluor 594 phalloidin (red), and microtubules by an antibody against α-tubulin (green). Scale bar indicates 10 *μ*m. (c) and (d) Left: representation of the absolute x–y position of cell organelles in relation to the middle of the T-shaped micropattern. Right: A zoomed-in view of the absolute positioning of organelles on the T-shape. The nucleus–centrosome axis (indicated by the dotted red arrow) orients toward the top right corner of the T-shape under the control condition (c), whereas this axis reorients toward the top left following the 10 nM Latrunculin A treatment (d). Analyzed cell number in control groups: N = 387 for nucleus, N = 109 for centrosome, N = 59 for Golgi apparatus, N = 59 for lysosome, N = 71 for mitochondria, N = 328 for actin, N = 148 for tubulin; analyzed cell number in Lat A groups: N = 610 for nucleus, N = 260 for centrosome, N = 109 for Golgi apparatus, N = 109 for lysosome, N = 86 for mitochondria, N = 501 for actin, and N = 155 for tubulin. Data in (c) and (d) are presented as mean ± SD in the wider image (left) and mean ± SE in the zoomed-in image (right).

Understanding the left–right positioning in cells with a demarcated front–rear polarity was made possible through the use of a T-shaped micropattern.^25^ Upon seeding C2C12 cells onto this micropattern, they extended into a triangular shape with the front–rear axis emanating from the top and bottom of the T-shape. Simultaneously, the left–right side of the cells was consequently established [Fig. 3(a)]. Fluorescence imaging permitted the precise localization of various cell organelles, including the nucleus, centrosome, Golgi apparatus, lysosome, mitochondria, actin filaments, and microtubules, to the front–rear and left–right axes [Fig. 3(b)].

Under the control condition, all the cell organelles were found predominantly on the front side of the mid-point of the T-shape (Table S1). Anticipatedly, this was bolstered by the enhanced attachment area offered by the head of the T-shape compared to the tail. When the left–right positioning bias was examined closely, the nucleus was typically located on the left side of the “T,” while the centrosome was situated at the front of the nucleus and to the right of the pattern [Fig. 3(c), Table S1]. This positioning permitted the cell to form an average nucleus–centrosome axis leaning toward the front-right direction of the T-shape. Interestingly, other organelles, such as lysosomes, mitochondria, actin, and tubulin, resided on the left side of the T-shape, which suggests an increased cell activity on the left side [Fig. 3(c)].

Conversely, following a 10 nM Latrunculin A treatment, all organelles stayed polarized at the front, as expected. Notably, the average nucleus position shifted toward neutral regarding the left–right bias, and the centrosome was found toward the front of the nucleus but on the left side of the T-shape [Figs. 3(b) and 3(d); Table S1]. This alignment, in concert with prior observations, supports the claim that Latrunculin A does not alter the front–rear polarity of C2C12 cells but does reverse their chirality on a ring-shaped micropattern.^6^ Therefore, the formation of a leftward tilted nucleus–centrosome axis is also observed under Latrunculin A treatment [Fig. 3(d)]. A peculiar development was the movement of the Golgi apparatus, lysosomes, and mitochondria to the right side of the T-shape, indicating an increased rightward cellular activity (Table S1).

**FIG. 4.**
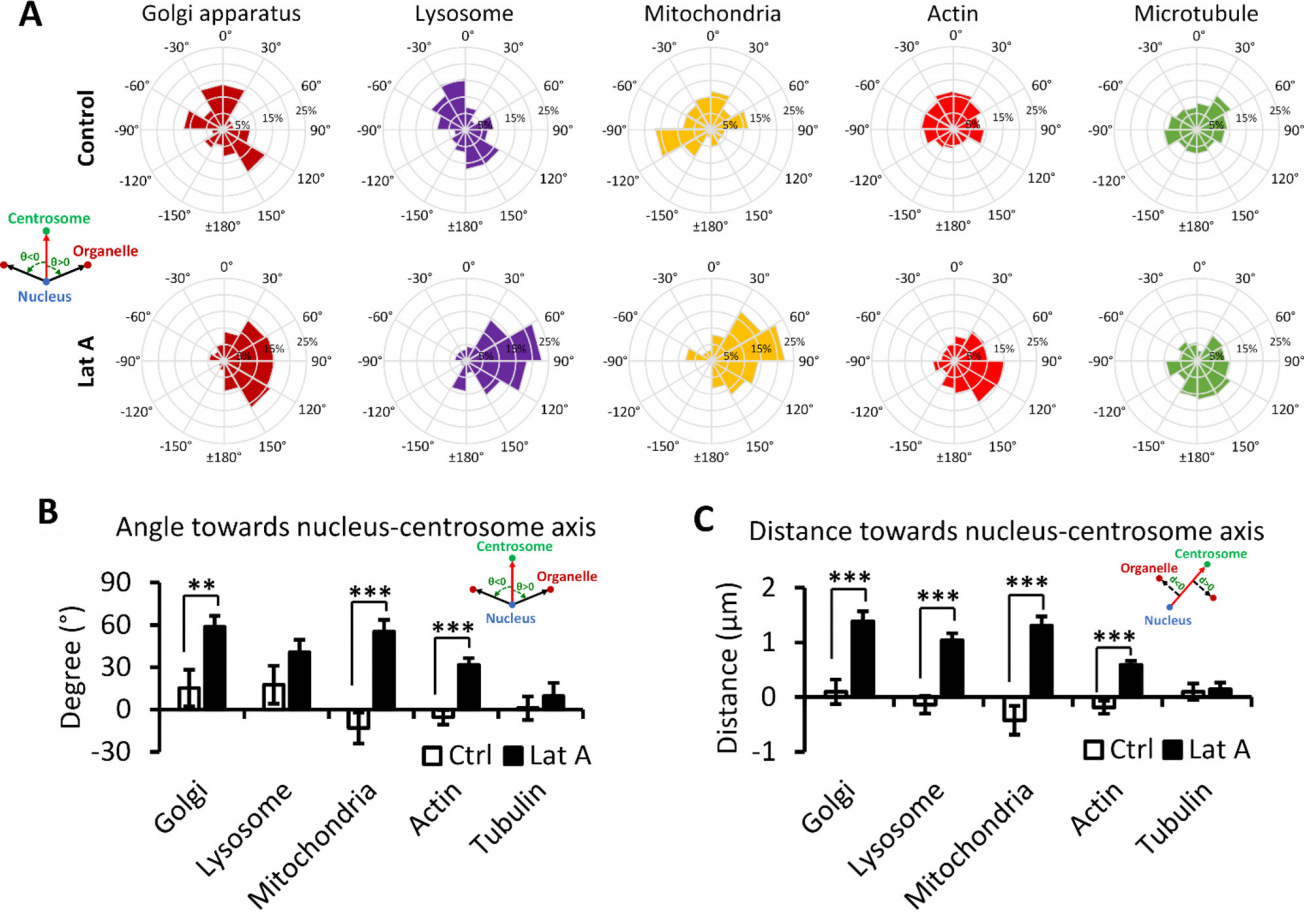
Examination of angular orientation of cell organelles in relation to the nucleus–centrosome axis. (a) The histogram of angular orientation of various cell organelles in relation to the nucleus–centrosome axis under the control condition or the 10 nM Latrunculin A treatment. A clockwise rotational angle is positive and counterclockwise rotations are denoted as negative values. (b) The summarized results of angular orientation of cell organelles in relation to the nucleus–centrosome axis. (c) The distances of other cell organelles toward the nucleus–centrosome axis under the control condition or 10 nM Latrunculin A treatment. Distances on the left side of the axis are represented as negative values, while those on the right are positive. Analyzed cell number in control groups: N = 59 for Golgi apparatus, N = 59 for lysosome, N = 71 for mitochondria, N = 328 for actin, N = 148 for tubulin; analyzed cell number in Lat A groups: N = 260 for centrosome, N = 109 for Golgi apparatus, N = 109 for lysosome, N = 86 for mitochondria, N = 501 for actin, and N = 155 for tubulin. Data in (b) and (c) are shown as mean ± SE. The asterisks stand for significant differences between the experimental group and the control: “^*^” indicates P < 0.05, “^**^” indicates P < 0.01, and “^***^” indicates P < 0.001, as determined by one-way ANOVAs with the Tukey method between groups.

Since the nucleus–centrosome axis is regarded as the front–rear polarity axis of a cell, we further investigated the positioning of other organelles to the nucleus–centrosome axis. Results revealed that on average, the positions of the lysosome, mitochondria, and actin are on the left side of the axis under the control condition; however, all organelles are located on the right side of the axis with Latrunculin A treatment [Figs. 3(c) and 3(d); Table S1]. Moreover, organelles, including the Golgi apparatus, lysosome, mitochondria, and actin, exhibit a significant clockwise angular orientation toward the nucleus–centrosome axis [Figs. 4(a) and 4(b)]. It is inferred that during cell migration, the nucleus–centrosome axis may lead the cell front, while the left–right biased positions of other cell organelles could indicate the cell turning direction. Notably, cell organelles become more dispersed following Latrunculin A treatment compared to the control condition. The Golgi apparatus, lysosome, and mitochondria's distance toward the nucleus–centrosome axis dramatically increase under the Latrunculin A treatment [Fig. 4(c)].

### Latrunculin A changes the positioning of cell organelles related to the cell nucleus

**FIG. 5.**
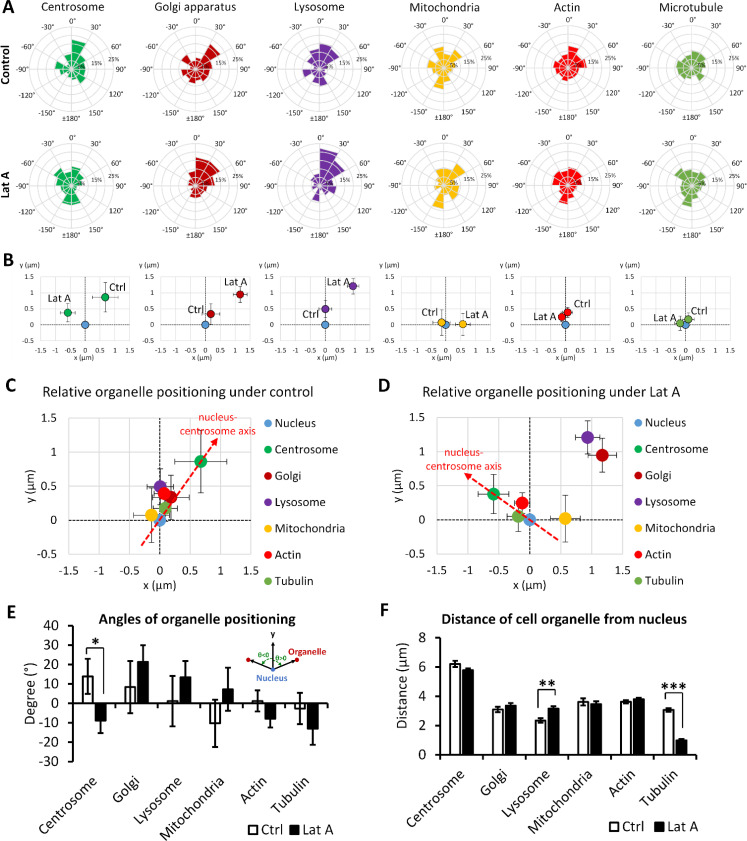
Analysis of the relative positioning of various cell organelles in relation to the cell nucleus. (a) The histogram of angular orientation of different cell organelles relative to the cell nucleus. A clockwise rotational angle from the front–rear axis of the T-shape (y-axis) is positive and counterclockwise rotations are denoted as negative values. (b) The average centroid of the centrosome, Golgi apparatus, lysosomal vesicles, mitochondria, actin filaments, or microtubules relative to the cell nucleus, in both control and 10 nM Latrunculin A treatment conditions. (c) and (d) Summary of the aggregated angular positioning of centroids of all analyzed cell organelles relative to the cell nucleus under the control condition or 10 nM Latrunculin A treatment. (e) The angular orientation of different cell organelles relative to the nucleus, where a clockwise rotational angle from front–rear axis of the T-shape (y-axis) is positive and counterclockwise rotations are denoted as negative values. (f) The absolute distances of different cell organelles away from the cell nucleus under the control condition or 10 nM Latrunculin A treatment. Analyzed cell number in control groups: N = 109 for centrosome, N = 59 for Golgi apparatus, N = 59 for lysosome, N = 71 for mitochondria, N = 328 for actin, N = 148 for tubulin; analyzed cell number in Lat A groups: N = 260 for centrosome, N = 109 for Golgi apparatus, N = 109 for lysosome, N = 86 for mitochondria, N = 501 for actin, and N = 155 for tubulin. Data in (b)–(f) are shown as mean ± SE. The asterisks stand for significant differences between the experimental group and the control: “^*^” indicates P < 0.05, “^**^” indicates P < 0.01, and “^***^” indicates P < 0.001, as determined by one-way ANOVAs with the Tukey method between groups.

The cell nucleus is commonly considered the center of the cell. This largest organelle oversees protein synthesis and regulates various cellular activities, spanning growth, metabolism, migration, and reproduction. In this study, the nucleus of the C2C12 cell is slightly at the left side of the cell body under control condition, while under the Latrunculin A treatment, it switched to a neutral place at the T-shape [Figs. 3(c) and 3(d)]. Additionally, the nucleus serves as a central hub for other organelles. The positioning of the nucleus may exert a significant influence over various cellular systems with different organelles, including the organization of the cytoskeleton, cellular signaling, transcriptional control, etc.^26^ Therefore, we performed an analysis of organelle positioning biases related to the cell nucleus to gain insight into the directional cellular activities that are governed by different organelles. A noteworthy shift was observed in the polarization of the centrosome, moving from the front-right to the front-left direction of the cell nucleus under the Latrunculin A treatment. In addition, there appeared to be a trend where the organelles, including the Golgi apparatus, lysosomes, and mitochondria, reoriented to the right side of the cell nucleus and moved further from it [Figs. 5(a)–5(d); Table S1]. Except for centrosome positioning, however, this shift induced by Latrunculin A treatment did not result in a significant difference when assessing the angular orientation [Fig. 5(e)].

The center of the actin filaments resides at the front side of the nucleus, making only a minor shift with Latrunculin A treatment. Furthermore, its average position is strikingly close to the nucleus [Fig. 5(b)]. This suggests that the actin centroid moves in unison with the cell nucleus either toward the left (control) or right (Latrunculin A). The tubulin centroid maintains its position on the same side as the centrosome toward the nucleus, under either control or Latrunculin A treatment. This shared behavior implies a combined movement of the centrosome and tubulin [Fig. 5(b)]. Taking into consideration the non-directional distance between these organelles and the cell nucleus, it is worth noting that when treated with Latrunculin A, lysosomes moved further away from the cell nucleus, whereas the tubulin filament centroid moved closer to the nucleus [Figs. 5(b) and 5(f)].

### The leading edge of the cell exhibits an asymmetrical pattern in metabolic activity and migration direction

**FIG. 6.**
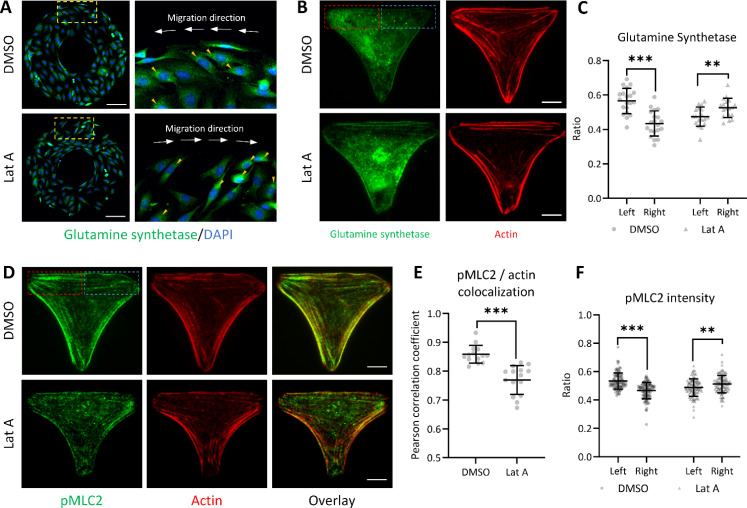
Asymmetrical distribution of glutamine synthetase and pMLC2 in C2C12 cells. (a) Fluorescent images display the asymmetric positioning of cell organelles near the border of the ring-shaped micropattern under the control condition and with 10 nM Latrunculin A treatment. Glutamine synthetase and nuclei in C2C12 cells were labeled with AF488-conjugated glutamine synthetase (green) and DAPI (blue), respectively. Higher magnification (40×) images on the right, of areas highlighted by yellow dashed boxes on the left, were captured to show detail. Small yellow arrows indicate areas of concentrated glutamine synthetase staining significantly positioned toward the predicted cell migration direction. (b) Fluorescent images of the single C2C12 cell labeled with glutamine synthetase (green) and actin (red) on the T-shaped micropattern. (c) A summary of the total staining intensity in the front-left [red dashed box in (b)] and the front-right [blue dashed box in (b)] regions for the T-shaped cell samples. (d) Fluorescent images of the single C2C12 cell labeled with pMLC2 (green) and actin (red) on the T-shaped micropattern, under control and with Latrunculin A treatment. (e) The colocalization of pMLC2 and actin filaments as shown in (d). (f) A summary of the total pMLC2 staining intensity in the front-left [red dashed box in (d)] and the front-right [blue dashed box in (d)] regions for the T-shaped cell samples. The dashed boxes in (b) and (d) have dimensions of 27 × 11 *μ*m^2^. Scale bar in (b) and (d) indicates 10 *μ*m. Data in (c), (e), and (f) are shown as mean ± SD. The asterisks stand for significant differences between the experimental group and the control: “^*^” indicates P < 0.05, “^**^” indicates P < 0.01, and “^***^” indicates P < 0.001, as determined by one-way ANOVAs with the Tukey method between groups.

The asymmetrical organization of cell organelles within C2C12 cells may result in a left–right bias in cellular activity. As the most abundant amino acid required by mammalian cells, glutamine synthesis is catalyzed by the glutamine synthetase. The presence and distribution of this enzyme not only reflect its metabolic activity of cells but also, potentially, the directional bias in cellular function and behavior.^27^ At the border region of the ring-shaped micropattern, we observed significant clustering of glutamine synthetase staining at the leading edge of cells pointing toward the counterclockwise or clockwise direction under the control condition or Latrunculin A treatment [Fig. 6(a)]. Thus, by utilizing the T-shaped micropattern model, we investigated the left–right cellular metabolic activity by imaging and quantifying the distribution of glutamine synthetase in individual cells [Fig. 6(b)]. The results showed a significantly higher intensity of glutamine synthetase at the left-front region compared to the right-front region of the cell under the control condition; notably, this pattern inverted following Latrunculin A treatment [Fig. 6(c)]. This suggests an association between biased cellular metabolic activity and the asymmetrical positioning of cell organelles.

Furthermore, pMLC2 is a significant marker consistently located at the cell leading edge, playing a pivotal role in the formation of lamellipodia and direction of cell migration.^28^ It contributes to the contractility of the actin network, facilitating forward cell movement.^29^ Our results indicated that pMLC2 exhibits high colocalization with actin filaments in cells on the T-shaped micropattern under the control condition. However, this colocalization is significantly reduced following Latrunculin A treatment [Figs. 6(d) and 6(e)]. There was no notable colocalization observed between pMLC2 and other organelles investigated, including the Golgi apparatus, mitochondria, lysosomes, and microtubules (Fig. S4). Additionally, pMLC2 displayed an asymmetrical left–right distribution similar to that of glutamine synthetase at the front region of cells [Fig. 6(f)]. This asymmetric distribution suggests that cells with differing chirality exhibit directionally biased migration-related activity at the leading edge.

## DISCUSSION

**FIG. 7.**
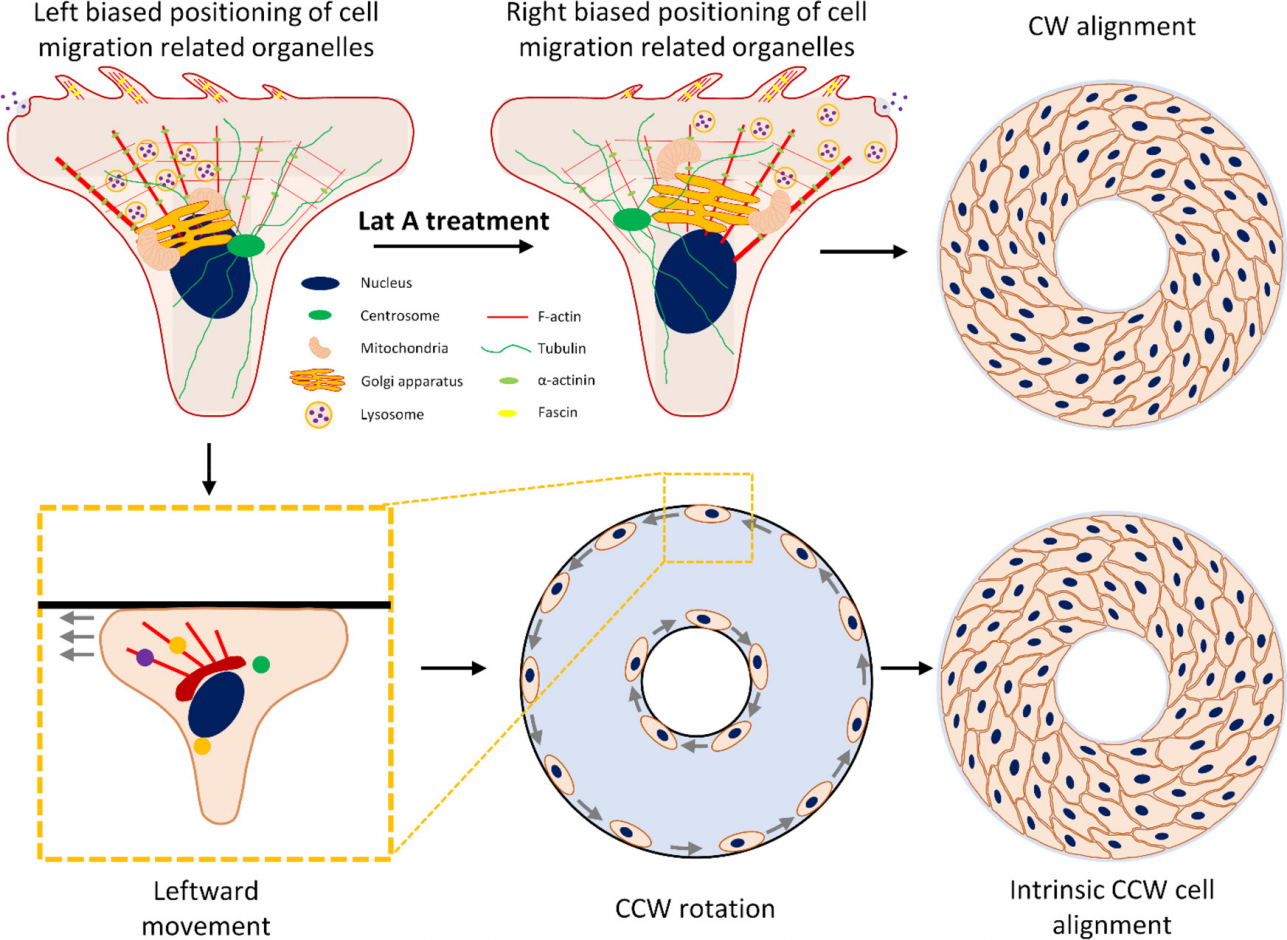
The potential mechanism of organelle positioning's influence on chiral multicellular morphogenesis. Under normal conditions in a myoblast cell, organelles associated with cell migration (e.g., lysosomes, mitochondria, actin, and the Golgi apparatus) typically align toward the front-left side of the nucleus–centrosome axis, while organelles involved in cell division (e.g., centrosome and tubulin) polarize to the opposite side of the cell. This unique positioning encourages a leftward cell movement at the pattern boundary. When observed across multiple cells, this leftward movement prompts a counterclockwise (CCW) multicellular rotation at pattern boundaries, subsequently inducing a CCW multicellular morphology. Contrastingly, when myoblast cells are treated with Latrunculin A, this preferential positioning pattern is reversed, resulting in rightward cell migration, leading to a clockwise (CW) multicellular morphology.

Biased migration toward the nucleus–centrosome axis was previously observed in HL-60 cells.^9^ We believe that this phenomenon is linked to cell chirality, as we have noted similar chiral migrations of different cell types at the periphery of ring-shaped micropatterns. We hypothesized that cell migration is closely associated with the active engagement of certain organelles. In this study, we observed a distinct polarization of front–rear cell polarity within a T-shape confined biosystem, transitioning from the front to the rear and from left to the right. The nucleus–centrosome axis exhibited a skew toward the tail–head direction of the T-shape with various cell organelles demonstrating a consistent bias in their positioning patterns. Specifically, when C2C12 cells exhibit a counterclockwise cell chirality under the normal condition, cell organelles—including lysosomes, actin, and mitochondria—tend to position themselves on the left side of the nucleus–centrosome axis. It indicates the potential direction of cell migration established by actin polymerization at the sites of filopodia or lamellipodia formation.^30,31^ Similarly, lysosomes actively receive and deliver substances in a dynamic pattern at the leading edge.^32^ The cell also strategically places mitochondria at the leading edge to meet the high energy demand and facilitate various processes.^33,34^ The organelle pattern could be further inferred from the higher demand for glutamine synthesis and actin filament contractility on one biased side of the cell, as indicated by the presence of glutamine synthetase and pMLC2 in front region (Fig. 6). Conversely, microtubules, which contribute to mitosis and the disassembly of focal adhesions,^35,36^ tend to be positioned on the right side of the cell (Fig. 7). This distinctive organelle positioning pattern may suggest a leftward prioritization in cellular metabolic activity and contractility for a biased cell migration, because the cell tends to generate more sensory chemical signals and develop new structures on the left side of the cell body, leaving the right side comparatively destructive.

When a cell is placed on a less confined adhesive micropattern, such as the ring-shaped micropattern, it is unable to migrate directly beyond the boundary. Instead, the cell displays the flexibility to navigate a turning direction, either left or right. In a less confined space, the biased positioning of organelles at the leading edge could profoundly alter the centroid of the cell body off from the nucleus-centrosome axis, a notion supported by prior studies.^18–22^ Particularly, when a C2C12 cell demonstrates a subtle left-biased organelle pattern, it triggers a leftward movement preference, resulting in a predominantly counterclockwise migration pathway for most cells on the micropattern boundary (Fig. 7).^6^

Contrastingly, Latrunculin A treated cells reveal a reversed cell chirality and organelle positioning pattern, signifying a correlation between cell migration and organelle arrangement (Fig. 7). Actin filaments are reported to initiate the breaking of left–right symmetrical structures within cells. Specifically, manipulating the expression of actin-polymerization-related proteins, including α-actinin-1, fascin-1, and profilin-1, has been found to alter the cell chiral behaviors in different cell types.^14,15,37^ Subsequentially, re-organization in cytoskeletal filaments may further alter organelle positioning, resulting in a pattern that potentially guides cell migration toward a different direction. Importantly, in a cell, organelles are complexly interconnected and organized by cytoskeletal proteins and organelle anchoring proteins, such as the linker of nucleoskeleton and cytoskeleton (LINC) complex which bridges the cell nucleus and cytoskeleton, and ANC-1, which have been implicated in the positioning of the endoplasmic reticulum and mitochondria.^38,39^ Additionally, the Golgi is connected to the microtubule network,^40^ which is further crosslinked with actin filaments.^41,42^ Given the crucial role of actin filaments in connecting these cell organelles, as well as the nucleus and plasma membrane, interference with actin polymerization by Latrunculin A could influence internal cellular organization and behavior even when cell is stationary. Therefore, organelle orientation may be affected by disrupting certain connections, particularly at the leading edge, by Latrunculin A. Again, the reorganization of cell organelle positioning might still follow the major functional needs of cells, such as contractility and metabolism.

Latrunculin A not only inhibits the polymerization of actin filaments but also promotes their depolymerization.^24^ The study indicates that following the treatment with Latrunculin A, although at a nanomolar range, there is a slight increase in the dispersion of organelles, in contrast to their more compact arrangement under the control condition. The observed dispersion, which could indicate a possible reduction in the reinforcement of linkage proteins or intermediate cytoskeletal filaments among organelles, may be a result of direct or indirect influence of Latrunculin A on enhancing the depolymerization of actin filaments and the LINC complex.^43^ Additionally, the actin network plays a critical role in cell stiffness and contractility.^44^ Disruption of this network by Latrunculin A may lead to a cell interior that is softer and less cohesive.^44^ Consequently, an expanded spatial separation between cell organelles could reasonably be anticipated following Latrunculin A treatment.

In an *in vitro* cultured cell model, the positioning of cell organelles often appears a certain degree of “randomness” due to the unpredictable factors of the biological environment. This study similarly reveals a level of randomness, particularly at the single-cell level where organelle positioning exhibits broad variation [Figs. 4(a) and 5(a)]. Despite an observed trend toward preferential directional positioning, this placement can be transient, influenced by the cell cycle or environmental modifications. This creates potential noise in organelle positioning, with further complications arising should the cell body not be confined by a micropattern. For instance, endothelial cells possess a strong clockwise bias under controlled conditions,^6,19^ yet analysis reveals merely a higher percentage of endothelial cells in a blood vessel orient their centroids toward the right side of the nucleus–centrosome than left.^19,20^ Nevertheless, on a tissue level composed of a large group of homogeneous cells, the impact of left–right biased cell behavior is amplified, with the bias of each cell potentially inducing a collective twist in tissue morphogenesis. This mirrors observations that, on a blood vessel wall, the cell nuclei align in a distinct, slanted direction toward the longitudinal axis of the blood flow.^45^ Furthermore, the observed preferential tissue morphogenesis during embryonic development stems from the left–right asymmetry of individual cells.^10,11,18^

Regardless, cells can establish left–right biased cellular activity to prompt asymmetrical behavior, such as alignment, rotation, or migration. In our study, the reversal of cell chirality could be signaled by the change in organelle positioning patterns. Understanding the positioning of cell organelles could offer significant insight for research in this field. Furthermore, this knowledge has the potential to improve predictions of cell migration and tissue morphogenesis, and it may serve as a valuable instrument for future biomedical applications.

## METHODS

### Cell culture, seeding, and treatment procedures

The mouse myoblast cell line (C2C12, ATCC, Manassas, VA, USA) was cultured and maintained within DMEM-High Glucose medium (DMEM-HG, Corning, NY, USA), supplemented with 10% fetal bovine serum and 100 U/ml penicillin-streptomycin. For cell chirality measurement, C2C12 cells were applied at a density of 100 k/ml in a total of 0.5 ml per sample on ring-shaped micropatterns; while for organelle positioning assessment, C2C12 cells were seeded at a density of 50 k/ml in a total of 0.5 ml per sample on T-shaped micropatterns. The samples were incubated for 10–15 min or until the cells adhered to the micropattern. Subsequently, the samples were transferred to either control or treatment medium and incubated overnight for the cell chirality test on the ring-shaped micropatterns or 3 h for the cell organelle positioning experiments on the T-shape. The 3-h period ensures that the majority of cells are completely spread out on the micropattern and have had 1 h to establish organelle polarity, while also maintaining a sufficient buffer before cell division occurs.

The cell chirality test utilized Latrunculin A (Cayman Chemical, Ann Arbor, MI, USA) concentrations of 0.5, 0.75, 1, 1.5, 2, 5, 10, 20, 50, and 200 nM. Other compounds used included Swinholide A (Cayman Chemical, Ann Arbor, MI, USA) at 5 nM, Cytochalasin D (Cayman Chemical, Ann Arbor, MI, USA) at 500 nM, and Paclitaxel (Cayman Chemical, Ann Arbor, MI, USA) at either 10 nM or 100 nM. For assessing cell organelle positioning on T-shaped micropatterns, 10 nM Latrunculin A was employed. Control groups were treated with 0.1% of DMSO in cell culture media.

### Cell viability assay

Cells were seeded at a density of 100k/ml in a total volume of 0.5 ml in 24-well plates and incubated with 1, 2, 5, 10, 20, 50, and 200 nM Latrunculin A for 24 h, with 0.1% DMSO in the cell culture media serving as a control. Subsequently, the cells were labeled with a Live/Dead staining kit (Thermo Fisher Scientific, Waltham, MA, USA) following the manufacturer's instructions. Ten images were taken at randomized locations for each sample. Live and dead cell numbers were counted using ImageJ (NIH). The cell viability was calculated as follows: *N*_Live_/(*N*_Live_ *+ N*_Dead_).

### Microcontact printing

**FIG. 8.**
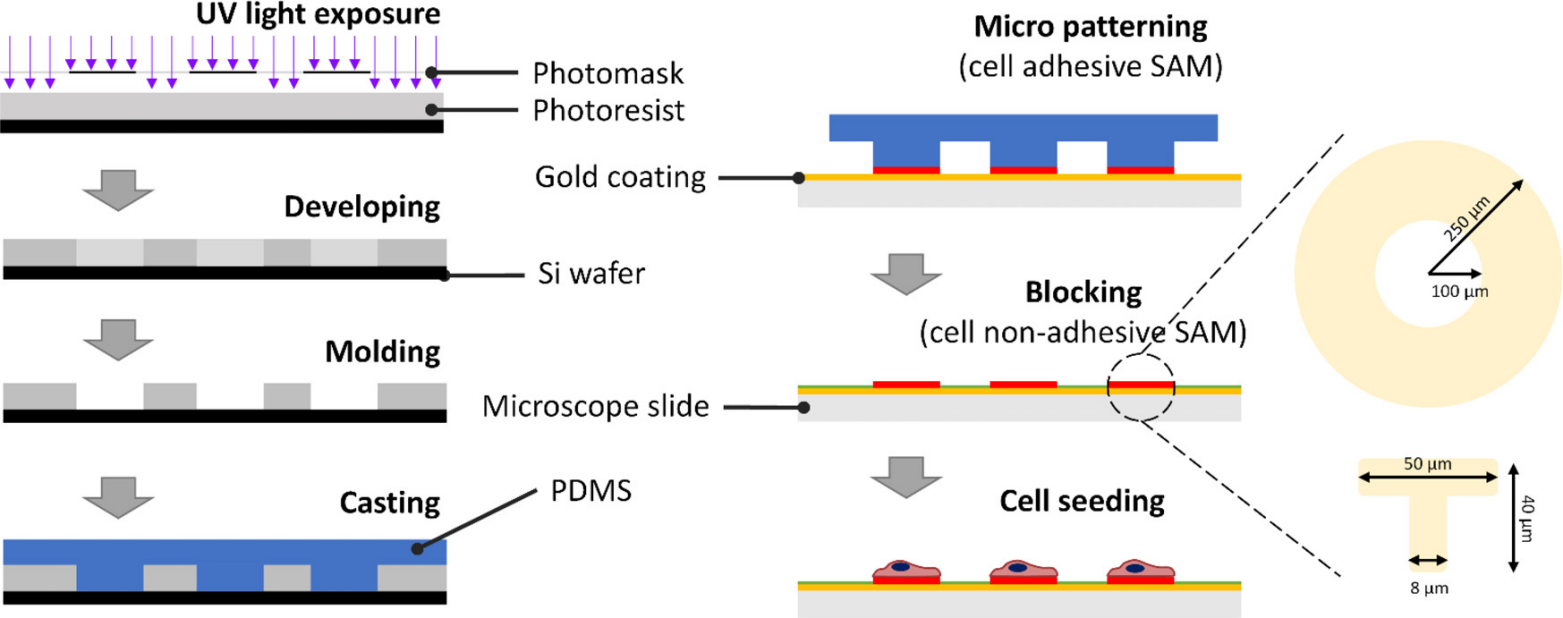
A schematic illustration of two processes: the fabrication of a micropatterned negative mold on a silicon wafer employing lithography and the establishment of cell adhesive micropatterns using PDMS stamps cast from the silicon wafer mold.

Cell adhesive micropatterns were generated using contact printing as described in our previous study.^6,19^ Briefly, a photomask was created with the ring-shaped feature (200 *μ*m inner diameter and 500 *μ*m outer diameter) or “T” shaped feature (CAD/Art Services, Bandon, OR, USA) (Fig. 8). SU-8 films were created on 100 mm Si wafers via photolithography. The polydimethylsiloxane (PDMS) stamp was then molded by incubating PDMS and the curing solution mixture (Krayden, Denver, CO, USA) at a ratio of 10:1 on the wafer. Then, Ti-Au coated glass slides (LGA Thin Films, Santa Clara, CA, USA) were printed with 1-octadecanethiol (MilliporeSigma, St. Louis, MO, USA) using the PDMS stamp [Figs. S5(a) and S5(b)]; subsequently, the printed slides were treated by the non-adhesive EG3 [HS-(CH2)11-EG3] (ProChimia Surfaces, Poland) for at least 3 h followed by a fibronectin (50 *μ*g/ml, MilliporeSigma, St. Louis, MO, USA) coating for 30 min to enhance the cell attachment (Fig. 8). T-shaped micropatterns of various sizes were fabricated and tested on C2C12 cells, and it was found that a size of approximately 50 *μ*m is optimal for the cells to completely spread out into a triangular formation [Fig. 3(b)]. Only T-shapes with a single, completely spread cell were used for imaging and analysis.

### Cell chirality analysis

When reaching confluency after 24 h, cell samples were fixed, and high-resolution phase-contrast images were captured using a Nikon Eclipse Ts2 microscope. The chirality analysis was performed using a custom-written MATLAB (MathWorks, Natick, MA, USA) program as described previously.^6,46^ The chirality of cells on each ring was determined as clockwise (CW, rightward), counterclockwise (CCW, leftward), or non-chiral (NC) based on the circular statistics of the distribution of cell alignment deviating from circumferential direction [Figs. 1(b)–1(d)].

### Immunofluorescence and cell organelle staining

Micropatterned cell samples were fixed with 4% paraformaldehyde, permeabilized with 0.2% Triton X-100/phosphate-buffered saline (PBS) for 15 min, blocked with 10% normal goat serum in 0.1% Triton X-100/PBS for 1 h, and incubated with primary antibodies at 4 °C overnight followed by a 1 h incubation at room temperature with corresponding secondary antibodies conjugated with either AlexaFluor 488 (green) or 594 (red). For samples that required cell nuclei staining, mounting was done in Fluoromount-G with DAPI (SouthernBiotech, Birmingham, AL, USA). Imaging was subsequently achieved through the Nikon Eclipse Ts2 fluorescence microscope using Nikon Plan Apo 40×/0.95. The primary antibodies applied against cellular components included rabbit anti-pericentrin (Thermo Fisher Scientific, Waltham, MA, USA) for addressing cell centrosomes, mouse anti-LAMP-1 (Santa Cruz Biotechnology, Dallas, TX, USA) for lysosomal vesicles, mouse anti-AIF (Santa Cruz Biotechnology, Dallas, TX, USA) for mitochondria, mouse anti-α-tubulin (Santa Cruz Biotechnology, Dallas, TX, USA) for microtubules, rabbit anti-glutamine synthetase (Thermo Fisher Scientific, Waltham, MA, USA), and rabbit anti-pMLC2 (Ser19, Cell signaling, Danvers, MA, USA) for phospho-myosin light chain 2. The Golgi apparatus was stained using either AlexaFluor 488 or 594 conjugated wheat germ agglutinin (WGA, Thermo Fisher Scientific, Waltham, MA, USA). Actin filaments were stained using AlexaFluor 488 or 594 conjugated phalloidin (Thermo Fisher Scientific, Waltham, MA, USA).

### Image analysis for cell organelle positioning

The coordinates for the front and rear points of the T shape [Fig. 3(a)], as well as the centroid of each type of cell organelles, were measured using the intensity-weighed centroid of the staining through Image J (NIH). These coordinates were subsequently entered into a customized MATLAB program (MathWorks, Natick, MA, USA) to calculate the front–back, left–right biases, orientation angle, and distances between each cell organelle relative to the front–rear axis of the T shape, the cell nucleus, or the nucleus–centrosome axis.

For quantifying glutamine synthetase and pMLC2 in single cells on the T shape, the total intensity of staining within dashed boxes measuring 27 × 11 *μ*m^2^ at the top-side margin of the T shape is shown in Figs. 6(b) and 6(d).

### Statistics

Data were presented as mean ± SD unless indicated otherwise. One-way ANOVA with the Tukey method was performed for multiple comparisons. Significant differences were accepted at P < 0.05.

## SUPPLEMENTARY MATERIAL

See the supplementary material for the screening of compounds known to effectively reverse cell chirality (Fig. S1); the viability result of C2C12 cells under the Latrunculin A treatment (Fig. S2); the fluorescent images showing the positioning of microtubules near the border of the ring-shaped micropattern (Fig. S3); the fluorescent images showing the co-staining of pMLC2 and various organelles (Fig. S4); the bright field images of the ring-shaped and T-shaped micropatterns used in this study (Fig. S5); and the numbers of organelles at the relative position of T-shape, nucleus–centrosome axis, and cell nucleus (Table S1).

## Data Availability

The data that support the findings of this study are available within the article and its supplementary material.
